# Towards Wearable Respiration Monitoring: 1D-CRNN-Based Breathing Detection in Smart Textiles

**DOI:** 10.3390/s25226832

**Published:** 2025-11-08

**Authors:** Tobias Steinmetzer, Sven Michel

**Affiliations:** Department of Therapy Sciences (II), Brandenburg University of Technology (BTU) Cottbus-Senftenberg, Platz der Deutschen Einheit 1, 03013 Cottbus, Germany; sven.michel@b-tu.de

**Keywords:** respiration, IMU sensor, deep learning, smart textile

## Abstract

Monitoring respiratory activity is a key indicator of physiological health and an essential component in smart textile systems for unobtrusive vital sign assessment. In this work, we present a one-dimensional convolutional recurrent neural network (1D-CRNN) for automatic classification of breathing activity from inertial data acquired by a smart e-textile of 59 subjects. The proposed method integrates convolutional layers for local feature extraction with recurrent layers for temporal context modeling, enabling robust segmentation of breathing and noise segments. The model was trained and evaluated using a stratified five-fold cross-validation scheme to account for inter-subject variability and class imbalance. Across different window sizes, the classifier achieved a mean accuracy of 0.88 and an F1-score of 0.92 at a window size of 2000 samples. The best-performing configuration for a single fold, reached an accuracy of 0.995 and an F1-score of 0.99. Furthermore, near-real-time feasibility was demonstrated, with a total processing time—including data loading, classification, segmentation, and visualization—of only 1.76 s for a 250 s measurement, corresponding to more than 100× faster than the recording time. These results indicate that the proposed approach is highly suitable for embedded, on-device inference within wearable systems.

## 1. Introduction

Monitoring respiratory rate and breathing dynamics is widely recognized as a crucial indicator of physiological state and overall health. In clinical settings, deviations in respiratory frequency can precede critical events such as respiratory failure, sepsis, or cardiovascular compromise [1]. In sports and occupational medicine, respiratory metrics provide sensitive markers of exercise tolerance, fatigue, heat stress, and exposure to hazardous environments [2,3]. Given the high global prevalence of respiratory diseases (e.g., asthma, COPD) and the need for long-term ambulatory surveillance, there is a strong incentive to develop unobtrusive, accurate, and robust respiration monitoring technologies [4].

Although conventional clinical methods such as spirometry and capnography deliver precise flow and gas measurements, they require bulky equipment and substantial user cooperation, limiting their suitability for continuous free-living monitoring [5]. Cesareo et al. deployed a multi-inertial measurement unit (IMU) system on the thorax and abdomen (plus a reference unit on a rigid body segment), effectively modeling the chest as a two-compartment system [6]. Wearable systems and smart textiles address this gap by enabling long-term, minimally intrusive recording of respiration-related body motion [7,8]. In particular, IMUs have emerged as a promising modality because they capture subtle chest and abdominal motion at a low cost and with minimal burden on the wearer [9,10]. IMU-based systems have been successfully applied to respiratory rate estimation, apnea detection, and in combination with other sensors, to suppress motion artifacts [7,9,11].

More recently, Kontaxis et al. used a five-IMU system (waist, arms, legs) to track vital signs during sleep, demonstrating adaptability across various physiological states [12]. Studies have noted that “inertial sensors open the door to the development of low-cost, wearable, easy-to-use breathing-monitoring systems” [7] and that IMUs provide a versatile method for collecting respiratory motion data [9].

Recent advances in wearable photonic devices highlight the potential of compact, body-worn sensors for continuous monitoring of vital signs. For example, Kuang et al. (2024) presented a smart photonic wristband capable of real-time acquisition of pulse wave signals during daily life, yielding high signal fidelity while maintaining low form-factor and power consumption [13].

This development underscores the broader shift from conventional, bulky clinical systems to unobtrusive, on-body platforms capable of long-term monitoring in free-living conditions. Embedding optical or inertial sensors into textiles and wristbands opens new pathways for continuous cardiovascular and respiratory assessment.

Despite this progress, several challenges remain. First, reliable phase segmentation (i.e., identification of inhalation and exhalation onsets and offsets) from wearable signals is still an open problem, particularly under motion or when sensor placement varies [8,14]. Second, many studies focus on respiration rate estimation or binary event detection (e.g., apnea vs. normal) but do not provide fine-grained segmentation of inhalation vs. exhalation, which is needed for richer physiological metrics (tidal timing, inspiratory/expiratory ratio). Third, robustness under dynamic conditions (walking, bending) and generalization across cohorts of different ages and clinical backgrounds are often insufficiently explored. Finally, although some systems implement on-device processing, most deep-learning solutions are developed and evaluated offline; achieving embedded, real-time inference in a power- and memory-constrained wearable remains challenging [4,8].

In this work, we address these gaps by presenting a feature-enriched 1D Convolutional-Recurrent Neural Network (1D-CRNN) for breathing detection and phase segmentation from an abdominal IMU integrated into a smart e-textile. Our contributions are summarized as follows:

- We provide a labeled dataset collected from 59 participants, including controlled static breathing sequences and dynamic gait trials, with IMU recordings sampled at approximately 330Hz and synchronized motion-capture reference data.
- We propose a compact 1D-CRNN architecture combined with targeted feature engineering (first-order differences and vector norms) yielding a 14-channel input per time step and improving discrimination between breathing and motion-induced noise.
- We evaluate the model using subject-independent GroupKFold cross-validation and extended fold analysis.
- We demonstrate practical feasibility: the full post-processing pipeline (data loading, segmentation, classification, visualization) processes a ∼240 s recording in ≈1.76 s on a standard laptop, indicating a strong potential for real-time operation.
- We present a case study showing that classified segments can be post-processed to extract interpretable respiratory parameters (inhalation, exhalation, and duration).

By situating our approach alongside recent IMU and textile-based respiration studies [4,7,8,10], we show that hybrid CNN–RNN models can achieve competitive performance while remaining suitable for embedded deployment. The remainder of the paper is organized as follows: Section 2 details the data acquisition, pre-processing, and model architecture; Section 3 presents cross-validation results, confusion matrices, and the case study; Section 4 discusses limitations and future directions; and Section 5 concludes the paper.

## 2. Methods

### 2.1. Dataset and Experimental Protocol

The dataset consists of recordings from 59 participants (17 male and 42 female) with an average age of 22.81 years (σ=1.82 years). Ethical approval for the study was obtained from the Ethics Committee of the Brandenburg University of Technology Cottbus–Senftenberg (Reference No.: EK2024-26). The experimental procedure was divided into two parts:

Static Breathing Test: Participants stood upright while performing a controlled breathing sequence. They first completed ten deep breaths (inhalation and exhalation) followed by ten normal breaths. The breathing movements recorded during this phase were fully annotated and labeled as the Breathing class for subsequent machine learning analysis.

Dynamic Gait Test: Participants performed a Timed Up and Go (TUG) test. The respiratory movements recorded during this task were labeled as the *Noise* class, as no consistent breathing patterns could be visually identified.

**Figure 1:**
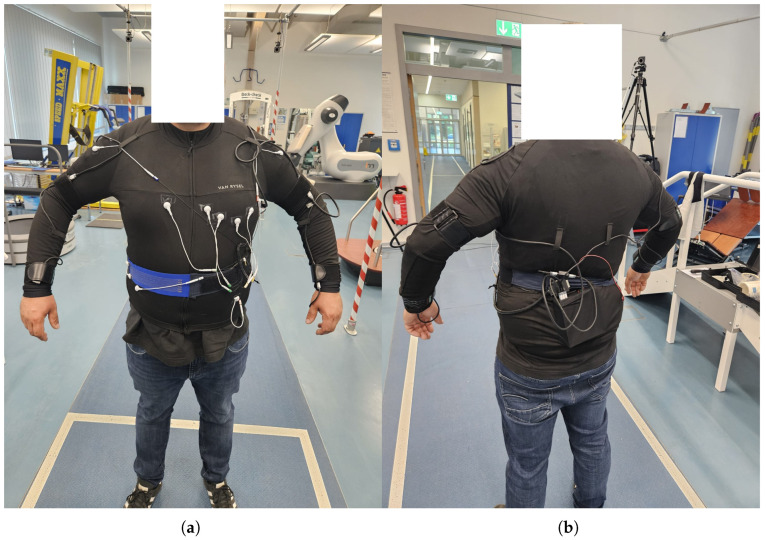
Prototype of the Smart e-Textile Singlet with functions for pulse, risk of falling, position, ECG, activity level, respiratory rate, oxygen saturation in the blood, core body temperature, and GPS. In (**a**) you can see the front of the singlet. (**b**) shows the back of the singlet.

The distinction between the Breathing and Noise classes serves as the foundation for developing and validating classification algorithms capable of detecting breathing movements under static conditions. Each participant was equipped with two Adafruit BNO085 IMUs  [15], as illustrated in Figure 1. One IMU was placed on the abdomen, approximately one hand width above the navel, while the second IMU was positioned on the spine, aligned parallel to the first sensor. For the present analysis, only the data from the abdominal sensor were used. The abdominal IMU was selected because the dominant respiratory motion is expressed in the abdominal wall, especially during diaphragmatic breathing. This placement provides the highest signal-to-noise ratio for detecting periodic breathing motion while minimizing interference from limb movement. Other IMU positions (e.g., spine and chest) were tested in preliminary recordings but yielded lower robustness due to stronger contamination from body motion and posture changes.

The IMU sensors were configured to record three-axis linear acceleration and three-axis absolute orientation (Euler angles) at a sampling frequency of approximately 330Hz. To validate the IMU data, ten reflective markers were additionally placed on the abdomen and spine to capture reference movements using a Vicon motion capture system [16].

**Figure 2:**
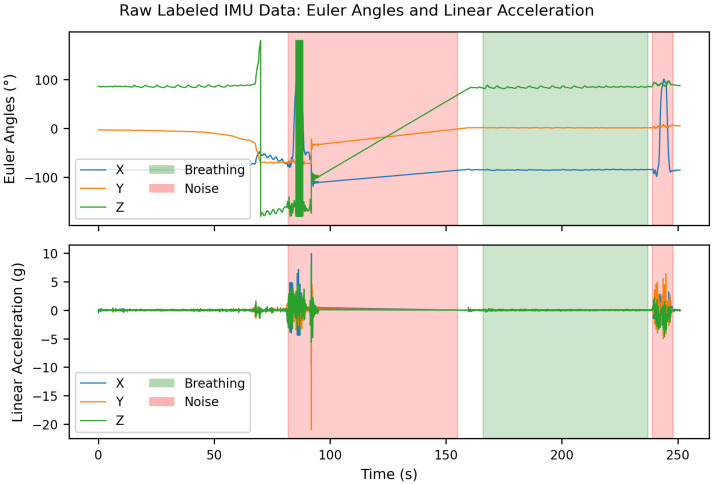
Raw IMU data recorded at 330 Hz: (**top**) Euler angles and (**bottom**) linear acceleration in three axes. Green areas indicate Breathing periods, while red areas represent *Noise*.

An example of the labeled raw data is shown in Figure 2. At the beginning of this recording, unlabeled respiratory segments are visible due to synchronization problems during the initialization of the Vicon motion capture system. These unlabeled areas were intentionally retained to later evaluate the robustness of the classifier to unlabeled or transitional data segments. Furthermore, a straight line is visible in the Euler angle signal approximately between 95 s and 160 s. The recording was stopped here to start approximately simultaneously with the Vicon system, in order to simplify subsequent data synchronization. This is why a gap exists in the data at this point.

#### Feature Engineering

For each segment, six primary sensor channels were used: three Euler angles (x,y,z) and three linear accelerations (x,y,z). To capture additional temporal and kinematic information, several derived features were computed:

- First-order differences in Euler angles and linear accelerations (ΔEuler,ΔAcc)
- Euclidean norms of acceleration and Euler orientation vectors

This procedure resulted in a feature vector of 14 dimensions per time step. All features were normalized using z-score normalization (mean = 0, standard deviation = 1) based on the training data only.

### 2.2. Machine Learning Model

The continuous IMU recordings were segmented using a sliding window approach with variable window lengths *w*∈ {250 (≈0.76 s), 500 (≈1.52 s), 1000 (≈3.03 s), 1500 (≈4.55 s), 2000 (≈6.06 s), 2500 (≈7.58 s)} samples and a fixed step size of 100 (≈0.3 s) samples. Each segment was assigned a label according to the annotated time intervals, resulting in two target classes: Breathing and Noise. To classify breathing and noise sequences, a one-dimensional convolutional recurrent neural network (1D-CRNN) was implemented using TensorFlow/Keras. Input sequences consisted of overlapping windows of lengths *w* samples, with 14 channels representing sensor axes.

Local features were extracted using two consecutive one-dimensional convolutional layers: the first with 32 filters (kernel size = 5) and the second with 64 filters (kernel size = 5), each followed by max pooling (pool size = 2). The resulting feature sequences were processed by an LSTM layer with 64 units to capture temporal dependencies. Dropout (*p* = 0.5) was applied for regularization. The classifier consisted of a fully connected dense layer with 32 units (ReLU activation) and a sigmoid output unit for binary classification.

### 2.3. Training and Evaluation

To ensure subject-independent evaluation, a five-fold GroupKFold cross-validation was performed. All data segments originating from the same subject were assigned to the same fold, preventing data leakage. This is to make sure that the same test subject is not included in the training and test data, as this would distort the validation process and result in an unrealistically good rating.

The dataset comprised 21,655 segments after applying the sliding window with a window size of 2000 samples (Noise = 5180, Breathing = 16,475). For a smaller window size of 250 samples, 24,876 segments were generated (Noise = 7157, Breathing = 17,719). Due to this class imbalance, unweighted training led to suboptimal model convergence. Therefore, a class-weighted training scheme was employed to compensate for the minority class.

The class weights were computed using the scikit-learn function compute_class_weight (‘balanced’, *…*), based on the formula

(1) $$weight_{i}=\frac{n_{total}}{n_{classes}}\cdot n_{i}$$

where $weight_{i}$ = denotes the weight of class i, $n_{total}$ the total number of training samples, $n_{classes}$ = 2 the number of classes, and $n_{i}$ the number of samples in class *i*.

The model was trained for 30 epochs with a batch size of 32 using the Adam optimizer and binary cross-entropy loss. Class imbalance was addressed via class weighting. Model performance was monitored on a validation set and all experiments were evaluated using 5-fold cross-validation across the different window sizes. After each fold, performance metrics including accuracy, precision, recall, and F1-score were computed. Additionally, confusion matrices were generated to visualize classification performance for each cross-validation iteration.

### 2.4. Inhalation and Exhalation Segmentation

Following the binary classification of breathing activity, a rule-based post-processing pipeline was applied to segment continuous breathing periods and estimate inhalation and exhalation durations. The entire procedure was implemented in Python 3.9 using NumPy, SciPy, and TensorFlow.

The continuous IMU signal was divided into overlapping windows of *w* with a step size of 100 samples (≈0.3 s). Each window was processed by the trained 1D-CRNN model, which outputs a probability of the breathing class. Predictions were binarized at a threshold of 0.5 and smoothed using a moving average filter of length five to reduce spurious transitions. Short isolated activations (<3 consecutive windows) were suppressed to enforce temporal consistency.

Consecutive windows classified as breathing were merged to form continuous breathing segments, each defined by its onset and offset time. These segments were used for subsequent phase-level analysis.

To obtain an orientation-independent measure of torso motion, the combined magnitude of the three Euler axes was computed as

(2) $${\left||v|\right|}_{2}^{2}=x^{2}+y^{2}+z^{2}.$$

This signal ${\left||v|\right|}_{2}^{2}$ was smoothed using a 0.5 s moving average to enhance respiratory oscillations. Inhalation and exhalation phases were derived by identifying local maxima (peaks) and minima in the smoothed signal using the find_peaks function from SciPy. Peaks corresponded to the end of exhalation (maximum thoracic inclination), while minima represented the end of inhalation. Durations between consecutive minima and maxima were used to compute the mean inhalation and exhalation times per segment, as well as the corresponding breathing duration.

## 3. Results

### 3.1. Classification and Model Performance

The proposed 1D-CRNN model was evaluated across multiple window sizes to assess the influence of temporal resolution on classification performance. Table 1 summarizes the mean and standard deviation of key metrics, including accuracy, precision, recall, and F1-score, for different temporal window sizes. A step size of 100 samples (≈0.3 s) and five-fold cross-validation were applied throughout the experiments.

A window size of 2000 samples (≈6.06 s) yielded the highest overall classification performance, with an average accuracy of 0.88 and an average F1-score of 0.92. This configuration provided an optimal balance between temporal context and model generalization.

### 3.2. Best-Performing Fold

**Figure 3:**
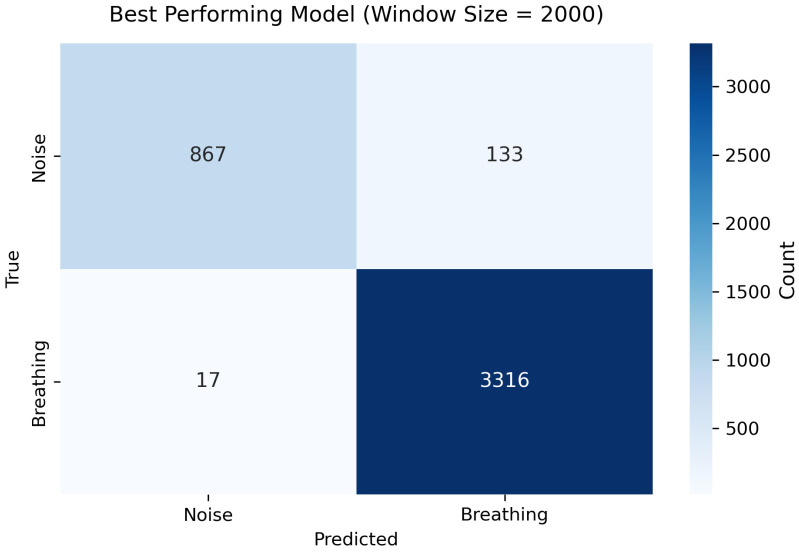
Confusion matrix of the best fold (Fold 2) for a window size of 2000 samples. The model shows strong discrimination between breathing and non-breathing (noise) segments, with only minor confusion at boundary regions.

The best result was achieved with a window size of 2000 samples (≈6.06 s), achieved an accuracy of 0.995, a precision of 0.996, a recall of 0.998, and an F1-score of 0.997. The corresponding confusion matrix is shown in Figure 3. The model demonstrated a high true positive rate for the Breathing class and a lower false positive rate for the Noise class, indicating robust discrimination between breathing and noise motion. Such near-perfect results highlight the robustness of the proposed architecture and confirm its suitability for real-time respiratory activity detection in smart textile applications.

**Figure 4:**
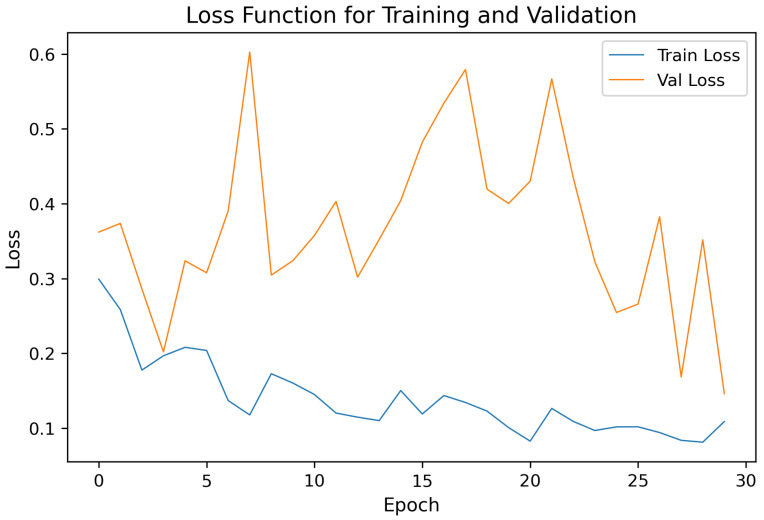
Training and validation loss curves of the best-performing fold (Fold 2) for a window size of 2000 samples (≈6.06 s). The model exhibits stable convergence.

Figure 4 illustrates the loss curve for the same fold (Fold 2) during training. The convergence behavior indicates stable optimization with minimal overfitting across epochs. The validation loss closely followed the training loss, confirming the robustness of the model, and the effectiveness of regularization techniques (dropout and batch normalization).

Overall, the 1D-CRNN demonstrated robust generalization across participants and effectively distinguished breathing movements under static conditions from motion-induced noise. The final model was retrained on the complete dataset using the best-performing configuration and stored for subsequent analyses and future deployment in real-time respiratory monitoring applications.

### 3.3. Case Study: Post-Classification Analysis of Breathing Segments and Performance

To further assess the interpretability and practical applicability of the proposed 1D-CRNN breathing classifier, a representative test recording was analyzed in detail. After classification, the detected breathing segments were post-processed to estimate key respiratory parameters, including the mean inhalation duration, mean exhalation duration, and total breathing cycle duration. For this analysis, the best-performing classification model described in Section 3.2 was used.

In addition, we recorded the timing of each classification step to evaluate near-real-time feasibility. In this analyzed sequence, a temporary interruption occurred between approximately 95 s to 160 s due to manual calibration of the motion capture setup. This resulted in a signal gap of about 65 s (≈21,450 samples), corresponding to a total effective recording length of 85,876 samples (≈260 s at 330Hz). The remaining 64,426 valid samples were used for windowing. The sliding-window procedure was implemented as $\left(N-Window_{size}\right)/Step_{size}$ = (64,426 − 2000)/100 = 62,426 ≈ 625 overlapping windows per sequence.

During inference, the term *classification step* refers to a single batch update rather than a single window. The model was evaluated with a batch size of 32, meaning that 32 windows were processed in parallel per classification step. Consequently, the 20 reported steps correspond to approximately 640 processed windows in total.

To evaluate near-real-time feasibility, we recorded the timing of each batch classification step. In the worst case, one batch step required ≈80 ms, leading to a total runtime of 1.6 s for all 625 windows. Including data loading, segmentation, and visualization, the complete pipeline required 1.76 s on our laptop. Considering a signal window of 6.1 s and a stride of 0.3 s, this corresponds to a near-real-time inference scenario with an update latency far below the signal acquisition rate. These results confirm that the proposed 1D-CRNN architecture operates at least 100 times faster than real time, indicating high suitability for embedded, on-device inference.

The proposed 1D-CRNN architecture comprises 47,713 trainable parameters, corresponding to a memory footprint of approximately 0.18 MB (32-bit precision). The estimated computational load per inference window is ≈0.1 million multiply–accumulate operations (MACs). These values indicate that the model is well within the computational and memory constraints of typical embedded microcontrollers and wearable systems.

In our intended embedded implementation, we utilize the Teensy 4.1 development board, as employed in our smart textile prototype. It is built around an ARM Cortex-M7 core running at 600 MHz, featuring 1 MB SRAM and 8 MB Flash [17]. The Cortex-M7 supports DSP extensions and single-cycle multiply/accumulate operations, enabling execution of tens to hundreds of millions of MACs per second, which provides ample computational headroom relative to the ≈0.1 million MACs required by our model.

On this basis, we characterize our classifier as capable of near-real-time on-device inference. With a window size of 2000 samples (≈6.06 s) and a stride of 100 samples (≈0.3 s), classification updates occur every 0.3 s, yielding a streaming-like update rate well below typical delays observed in wearable monitoring systems.

**Figure 5:**
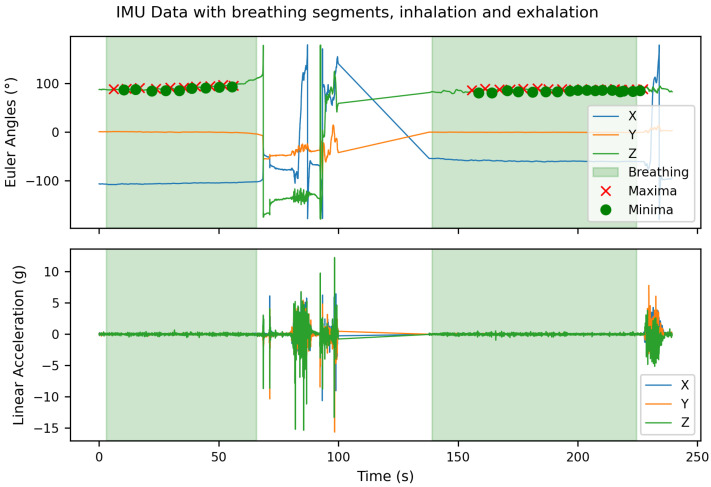
Visualization of the classified test sequence for one representative subject. The predicted breathing, inhalation, and exhalation is shown over time. The plot illustrates the ability of the proposed classifier to distinguish valid respiratory patterns from noisy or irregular intervals.

Table 2 summarizes the results for two consecutive segments of a complex test sequence. The Segments contain consistent breathing activity, with mean breathing durations of approximately 5.6 s and 4.2 s, respectively. These values correspond well to physiological expectations for resting respiration in young adults (approximately 12–16 breaths per minute). In contrast to Segment 2, Segment 1 contains unlabelled data, so it will be interesting to see how the model performs. Figure 5 visualizes the corresponding classification results for the same test subject. The time series plot highlights the predicted breathing (inhale/exhale) and non-breathing (noise) intervals. It demonstrates that the classifier reliably separates valid respiration cycles from segments containing motion artifacts or missing signal components. This case study illustrates that the proposed model not only enables accurate classification of breathing versus non-breathing periods but also facilitates the extraction of interpretable physiological parameters from continuous sensor recordings. Such post-classification analysis represents a promising step toward automated respiration monitoring in wearable systems.

## 4. Discussion

The results demonstrate that the proposed 1D-CRNN is an effective approach for discriminating breathing-related IMU signals from motion-induced noise under the defined experimental conditions. A window size of 2000 samples (≈6.1 s) provided the best trade-off between temporal context and classification stability, yielding high values of accuracy and F1-score (see Table 1 and the best-fold metrics in Section 3.2). This finding suggests that capturing at least one full respiratory cycle plus contextual neighboring samples improves the model’s ability to resolve breathing patterns from short, transient motion artifacts.

The confusion matrix for the best fold (Figure 3) indicates a high true positive rate and a low false positive rate for the Breathing class; most misclassifications occurred at transition regions where breathing overlapped with minor postural movements. This behaviour is consistent with the expected ambiguity at class boundaries and highlights an intrinsic limitation of hard binary labeling in regions that contain mixed or partial respiratory motion. The loss curves (Figure 4) show stable convergence and only minor divergence between training and validation loss, suggesting that the chosen regularization (dropout) and training strategy were effective in preventing overfitting on the available data.

Class imbalance was handled by computing class weights per training fold (mean weights across folds: Breathing = 0.702, Noise = 1.738), which improved sensitivity to the minority class and prevented performance degradation due to dominance of the majority class. Although weighting improved recall for the minority class, future work could explore complementary strategies such as targeted data augmentation, synthetic minority over-sampling, or focal loss to further increase robustness in highly imbalanced scenarios.

Several practical and methodological limitations should be acknowledged. First, the dataset comprises predominantly young adults (mean age 22.9 years), which limits immediate generalizability to older populations or patients with respiratory disorders. Second, only the abdominal IMU was used for the present analysis; additional sensor locations (e.g., chest, multiple abdominal sites) or sensor fusion (IMU + respiratory belt) might increase robustness, especially during dynamic tasks. Third, the Noise label currently aggregates all non-identifiable respiratory activity (including dynamic gait), so the model was not optimized for detecting breathing during vigorous motion; future studies should aim at multi-class annotation (e.g., breathing during low-motion vs. high-motion) to enable more granular classification.

From a deployment perspective, the optimal window length (≈6.06 s for 2000 samples) implies a trade-off between detection latency and classification reliability. For real-time monitoring, smaller windows reduce latency but may sacrifice accuracy; a sliding decision strategy (e.g., ensemble voting across overlapping windows) could mitigate this trade-off. Computational cost and memory footprint were moderate for the chosen 1D-CRNN.

Finally, the dataset included initial unlabeled segments due to synchronization issues with the Vicon system. While these segments were intentionally retained to evaluate classifier robustness to unannotated or transition data, they also underscore the importance of reliable synchronization and data quality in multimodal acquisition setups.

### 4.1. Performance Metrics and Real-Time Feasibility

Reported accuracies for binary breathing detection or apnea classification generally range from 88 to 95% [4,11]. Multi-class classification tasks tend to yield lower F1-scores (0.5–0.85), depending on the complexity of classes and class balance. SVM-based feature models on smaller datasets still show high accuracy (up to 97–99%) [18]. Real-time implementation is less common; most deep-learning studies rely on offline inference, though De Fazio’s device demonstrates that embedded firmware processing is feasible [8].

In this study, we presented a robust classification model for detecting breathing activity using inertial data from a smart e-textile system. The proposed 1D-CRNN achieved a mean accuracy of 0.88 and an F1-score of 0.90 across cross-validation folds. The best-performing configuration reached an accuracy of 0.995 and an F1-score of 0.99. These results are comparable or better to state-of-the-art approaches reported in the literature [4,8,11]. However, it should be noted that our evaluation protocol differs, as we employed a static breathing test rather than continuous or sleep-related monitoring tasks. Therefore, while the absolute performance values are similar, the contextual interpretation of these results must consider the specific experimental setup.

In addition to accuracy metrics, the computational efficiency of the proposed model was evaluated to assess its real-time feasibility. In the worst case, the runtime was approximately 100× faster than real time, underscoring the potential of the model for embedded, on-device inference within smart textiles. This measurement includes data loading, segmentation, and visualization on a standard laptop, demonstrating that the proposed system is well-suited for integration into embedded platforms.

### 4.2. Sensors and Data Acquisition

Most wearable studies rely on inertial sensors (accelerometers and gyroscopes) attached to the chest, abdomen, or back. Chang et al. used tri-axial IMUs sampled at 100 Hz to capture breathing movements [11]. De Fazio et al. developed a dual-IMU chest band that computes differential acceleration onboard, enabling real-time extraction of respiratory rate and phase durations [8]. Textile-integrated strain or piezoresistive sensors have also been explored. Ramos-Garcia et al. implemented a cover-stitched stretch sensor in a shirt and monitored breathing using FFT-based analysis [19]. While traditional respiration belts remain a reference modality, the trend in recent research favors lightweight, unobtrusive sensor systems. Study population sizes range from small laboratory tests (10–15 subjects) to larger cohorts; McClure’s dataset included N=100 participants [20], while Ryser et al. analyzed overnight recordings from 13 subjects using a single chest accelerometer [14].

Similar to related studies, our system employs a smart e-textile platform that integrates inertial sensors directly into a wearable garment. The presented respiratory analysis represents only one component of the overall sensing architecture. In combination with additional modules—such as ECG, skin temperature, blood oxygen saturation, pulse, GPS, and stretch sensors—the system enables a comprehensive assessment of the wearer’s physiological and physical condition. Future work will explore sensor fusion strategies to further enhance robustness and reliability across diverse daily-life scenarios.

### 4.3. Breath Segmentation and Rate Extraction

Several algorithms aim to detect complete inhalation–exhalation cycles or apnea events. De Fazio et al. reported excellent agreement (MAE ≤ 5%) for inhalation and exhalation duration estimation compared to a reference system [8]. Ryser et al. estimated respiration rate from chest acceleration signals with a mean error of about 1.8 breaths/min [14]. Other works classify individual events: McClure’s CNN model achieved per-second F1-scores between 0.86 and 0.95 across different breathing-related events [20]. These studies demonstrate that phase segmentation and respiratory rate estimation are achievable using single or dual IMUs under controlled conditions.

It is well established that the physiological breathing cycle in resting healthy adults typically spans approximately 3 to 5 s (corresponding to a respiratory rate of about 12–20 breaths per minute) [21]. In our experiments, we found that the highest classification performance occurred at a window length of approximately 6.06 s. This observation supports the conclusion that the classifier benefits from processing at least one full respiratory cycle and ideally slightly more to capture both inhalation and exhalation dynamics within a single segment. Thus, the results confirm that windowing strategies aligned with the biomechanical duration of the breathing cycle can enhance the discriminative power of time-series models for respiratory activity detection.

### 4.4. Context for the Proposed 1D-CRNN

In comparison, our proposed 1D-CRNN combines convolutional feature extraction with temporal modeling to identify breathing phases. This approach aligns with current trends in CNN–LSTM hybrid architectures, which have shown robust results in similar applications. Despite the unbalanced class distribution in our dataset, class weighting during training enabled reliable differentiation between breathing and motion-induced noise. The obtained F1-score and accuracy are comparable to state-of-the-art IMU-based methods (85–95%), while our implementation remains computationally lightweight and suitable for real-time execution. These findings confirm that IMU-based respiratory analysis using deep hybrid models is both feasible and competitive with other wearable sensor systems and may serve as a foundation for future multimodal sensor fusion approaches.

## 5. Conclusions

We presented a 1D Convolutional Recurrent Neural Network (1D-CRNN) for automatic detection of breathing activity from abdominal IMU recordings. By combining convolutional feature extraction with recurrent temporal modeling and a targeted feature engineering pipeline, the proposed system achieved robust subject-independent performance; the best configuration (window size = 2000 samples ≈ 6.06 s) yielded the highest overall F1-score across cross-validation folds (Table 1), and the best fold demonstrated excellent precision and recall (see Section 3.2 and Figure 3). The proposed model achieved high classification accuracy and real-time capability, indicating its potential for unobtrusive health monitoring applications.

Key contributions of this work include (i) a complete acquisition and labeling protocol with IMU and motion-capture validation, (ii) a feature-enriched CRNN architecture tailored for respiratory signal detection, and (iii) an extensive cross-validation strategy (GroupKFold and extended fold evaluation) to ensure subject-independent assessment. The proposed approach not only discriminates breathing from motion-induced noise but also enables post-classification extraction of physiologically relevant metrics (e.g., inhalation/exhalation durations), as illustrated in the case study (Table 2, and Figure 5).

Future work will focus on four main directions: (1) fusing inertial measurement data with stretch sensor signals placed on the abdomen to improve the robustness of breathing phase detection; (2) validating the extracted respiratory signals against certified medical reference devices to precisely assess the segmentation accuracy; (3) evaluating the method on more diverse populations and clinical cohorts to ensure generalizability; and (4) extending the annotation taxonomy to include breathing under different motion intensities, thereby distinguishing between static and dynamic activities (e.g., gait or posture transitions) to better quantify model robustness under motion.

In addition, coupling the proposed respiration classifier with an external activity recognition module (e.g., from a smartphone or smartwatch) could allow context-aware, energy-efficient operation of the smart textile system [22]. Such a multimodal approach would enable adaptive model activation only during relevant conditions (e.g., resting or sitting phases), reducing on-device computation and power consumption.

In summary, the proposed system represents a promising step toward unobtrusive, continuous respiratory monitoring using wearable IMU sensors and has potential applications in ambulatory health monitoring, sleep assessment, sport, and fall-risk-aware respiratory surveillance.

## Figures and Tables

**Table 1:** Classification performance of the proposed 1D-CRNN model for different window sizes (step size = 100 (≈0.3 s), $n_{splits}$ = 5). Values are reported as mean ± standard deviation. The best performance was achieved for a window size of 2000 samples (≈6.06 s) (highlighted in bold).

Window Size	Accuracy	Precision	Recall	F1-Score
250	0.859 ± 0.026	0.924 ± 0.039	0.875 ± 0.043	0.897 ± 0.023
500	0.822 ± 0.068	0.922 ± 0.021	0.825 ± 0.096	0.867 ± 0.055
750	0.841 ± 0.021	0.929 ± 0.023	0.846 ± 0.035	0.885 ± 0.016
1500	0.870 ± 0.035	0.925 ± 0.027	0.898 ± 0.060	0.910 ± 0.028
**2000**	**0.880 ± 0.029**	0.925 ± 0.056	**0.922 ± 0.062**	**0.920 ± 0.022**
2500	0.856 ± 0.078	0.925 ± 0.062	0.889 ± 0.093	0.903 ± 0.056

**Table 2:** Example of classified test data used for post-processing analysis. Detected breathing segments were analyzed to estimate mean inhalation, exhalation, and total breathing durations.

Segment	Breaths	Inhalation (s)	Exhalation (s)	Breathing Duration (s)
1	10	3.974	1.589	5.563
2	18	2.402	1.802	4.204

## Data Availability

The data presented in this study are available on request from the corresponding author. The data are not publicly available due to ethical restrictions and institutional data protection policies, as well as to prevent potential misuse by commercial entities. Data can be shared for academic research purposes upon reasonable request.
